# Solving molecular compounds from powder diffraction data: are results always reliable?

**DOI:** 10.1107/S2052252522006571

**Published:** 2022-06-30

**Authors:** Angela Altomare

**Affiliations:** a Institute of Crystallography, CNR, Bari, Via Amendola 122/O, Bari 70126, Italy

**Keywords:** structure solution, powder diffraction, ambiguous structure determination, low crystallinity, pair distribution function, solid-state nuclear magnetic resonance

## Abstract

Commentary is given on a paper [Schlesinger *et al.* (2022). *IUCrJ*, **9**, 406–424.] reporting on ambiguous structure determination from powder data using four different structural models of 4,11-difluoroquinacridone with similar X-ray powder patterns.

Powder X-ray diffraction (PXRD) is a powerful tool for solving microcrystalline powder structures. Writing in **IUCrJ** , Schlesinger *et al.* (2022 ▸) report intriguing results demonstrating that, in the case of poor-quality PXRD data, starting from one experimental pattern, we can obtain different structure solutions, all crystallochemically plausible and satisfying the reliability criteria currently adopted in the solution process. This unexpected situation suggests adopting a more critical attitude towards structure determination from powders.

Crystallographic study is the leading scientific approach for recovering unambiguously the arrangement of atoms in a crystalline system. In the case of a single crystal, the structure determination is rarely affected by errors because the quality of the experimental diffraction data is usually good. However, in the powder case, the structural study faces several, and often concurrent, problems due to peak overlap, background noise, preferred orientation, limited quantity, poor quality of diffraction data, *etc.* Nevertheless, structure solution from powders has registered an increasing success over the years (*cf.* Fig. 1 ▸). Thanks to valuable methodological advances and high-performance computational resources (David, 2019 ▸; Altomare *et al.*, 2019 ▸; Černý *et al.*, 2019 ▸; Gilmore *et al.*, 2019 ▸), we are now able to quite easily solve small molecular structures, especially if Bragg peaks in the diffraction patterns are sharp and the background noise is low. In particular, global optimization (GO) methods have become very popular to the point that they are sometimes preferred to direct methods, even when the latter can be successfully applied. In addition to the determination of unit-cell parameters and space group, the solution process through GO methods requires comprehension of the expected molecular geometry. The GO methods randomly move a starting structure model around the cell, in accordance with the assumed symmetry, adjusting its position, orientation and conformation (degrees of freedom, DoF) with the aim of reaching the global minimum of a cost function (CF, based on agreement between the observed and calculated profiles) and providing the correct solution. The solution process is completed by Rietveld refinement, followed by a careful crystallochemical check of the structure solution. Validation with dispersion-corrected density functional theory (DFT-D) calculations is recommended (van de Streek & Neumann, 2014 ▸). Finally, the corresponding CIF (crystallographic information framework) file is submitted to a crystal structure database (*cf.* Fig. 1 ▸).

Schlesinger *et al.* focus their PXRD investigation on the unknown crystal structure of 4,11-di­fluoro­quinacridone (C_20_H_10_N_2_O_2_F_2_, DFQ), a non-commercial organic pigment derivative of quinacridone. With a rigorous and deep analysis, they prove that the DFQ structure is ambiguously solved if the solution process is carried out by a GO method followed by Rietveld refinement. At the same time, they demonstrate that a suitable combination of complementary methods can overcome this ambiguity and move towards the correct solution.

The DFQ poor-quality experimental diffraction pattern, collected on a laboratory X-ray powder diffractometer, presents only a few sharp and some broad Bragg peaks with severe overlap. Owing to a low level of crystallinity, the inadequate quality of this pattern cannot be improved upon by using synchrotron radiation. As things stand, the determination of unit-cell parameters by the most widely used indexing software is unattainable and the structure solution of DFQ can be classified as challenging.

Schlesinger *et al.* try to solve the DFQ structure by using the GO-based computing program *FIDEL-GO* (Habermehl *et al.*, 2022 ▸), which has been specifically developed for structure determination from unindexed data. The rationale of *FIDEL-GO* is the global fit between simulated and experimental powder data: about 21 million random starting structure models compatible with the expected molecular geometry, located in cells with random parameters and different space groups, are submitted to the optimization process; unit-cell parameters, molecular position and orientation, and selected internal DoF are fitted simultaneously to the powder pattern; a similarity measure based on cross-correlation functions allows the comparison of simulated and experimental data. An automatic Rietveld refinement, a DFT-D geometry optimization and a user-controlled Rietveld refinement complete the solution process.

Four crystallographically different solutions, all worthy of being published, are obtained thus making uncertain the principle of structure unambiguity: they are all chemically reasonable (check of molecular packing, intermolecular distances, short intermolecular contacts); they are all compatible with the experimental diffraction pattern, and they surprisingly end with acceptable low *R*-values from Rietveld refinements; they all pass the *CheckCIF* test with trivial alerts. From an extensive examination of topology and Rietveld fits, only a slight preference for two of the solutions comes to light. Which one of them is correct?

Examples of incorrect published structures and structure ambiguity from PXRD are reported in the literature, but Schlesinger and colleagues go beyond this and accomplish a comprehensive analysis of the four structure candidates. They accurately compare the candidates and disclose why they are all compatible with the same experimental profile. Moreover, they reveal structure disorder by inspecting peak broadening.

It is significant that the authors solve the crux of ambiguity with the aid of additional analyses: (*a*) intramolecular and intermolecular examination through extensive pair distribution function (PDF) refinements (Schlesinger *et al.*, 2021 ▸) using synchrotron data collected at both room temperature and 173 K; (*b*) evaluation of colour; (*c*) lattice-energy minimizations using force fields (Dassault Systemes, 2008 ▸) by optimizing molecular geometry and lattice parameters; (*d*) lattice-energy minimizations by DFT-D (Neumann *et al.*, 2008 ▸; Giannozzi *et al.*, 2017 ▸) by optimizing atomic positions and lattice parameters and calculation of the root mean square Cartesian deviation (RMSCD) of non-hydrogen atoms (van de Streek & Neumann, 2010 ▸); (*e*) solid-state NMR study with measurements of ^13^C, ^1^H and ^19^F spectra.

Overall, this insightful study reveals that structures solved from limited-quality PXRD data can be questionable if not supported by a multi-methodological solution strategy, as well as providing the correct structure of DFQ. The authors interestingly demonstrate that the application of the PDF fit method for structure refinement is promising.

A reasonable question arises from this work: can the combination of methods applied by Schlesinger *et al.* be generalized? Does this combination remove ambiguity in other similar cases? The issue remains open.

*The answer to the question in the title is yes, even in the case of low crystallinity, but a critical inspection of results must be carried out every time the solution process is executed by using methods based only on the best fitting of the experimental PXRD pattern.*

## Figures and Tables

**Figure 1:**
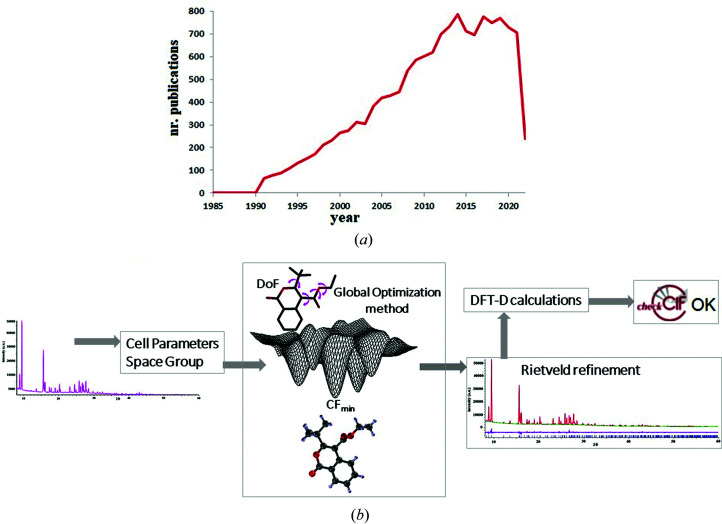
Progress in structure solution from PXRD data. (*a*) Approximate numbers of publications involving structure solution from powder diffraction (source: Web of Knowledge search, June 2022). (*b*) Steps of a crystal structure solution process from PXRD through global optimization methods.
